# Cenobamate Plasma Levels in Patients with Epilepsy: Correlation with Efficacy and Tolerability?

**DOI:** 10.3390/jcm13102757

**Published:** 2024-05-08

**Authors:** Bernhard J. Steinhoff, Dimitra Georgiou, Daniel Dietmann, Tassanai Intravooth

**Affiliations:** 1 Kork Epilepsy Center, Kehl-Kork, 77694 Kehl, Germany; dgeorgiou@epilepsiezentrum.de (D.G.); ddietmann@epilepsiezentrum.de (D.D.); tintravooth@epilepsiezentrum.de (T.I.); 2 Medical Faculty, University of Freiburg, 79104 Freiburg im Breisgau, Germany

**Keywords:** epilepsy, cenobamate, plasma levels, therapeutic drug monitoring, effectiveness, correlation

## Abstract

**Objective:** Cenobamate is approved by the European Medicine Agency for the treatment of adult patients with epilepsy (PWEs) with ongoing focal-onset seizures despite appropriate treatment with at least two established antiseizure medications. Pivotal trials and post-marketing real-world observational studies suggest high efficacy with unusually high seizure-free rates. The authors sought to investigate the plasma levels of cenobamate under steady-state conditions in seizure-free versus non-responding PWEs, and in PWEs who experienced adverse events versus those who did not. **Methods:** Blood samples were collected from adult PWEs who were treated with adjunct cenobamate under steady-state conditions. Daily doses, concomitant medications, efficacy, and tolerability were assessed. The plasma cenobamate levels of seizure-free versus non-responding PWEs and between PWEs with and those without clinical adverse events were compared. **Results:** Samples from 101 PWEs were included. Thirty-six PWEs were seizure-free and 65 were non-responders. In 31 PWEs, adverse events were apparent, whereas in the remaining 70, no tolerability issues were reported. A linear correlation was found between the daily doses (range: 100 mg–400 mg) and the plasma levels (3.8 mg/L–54.6 mg/L). Neither the daily doses nor the plasma levels differed significantly between the investigated subgroups. The main reason for this result was that the individual therapeutic ranges varied widely: seizure freedom and adverse effects were observed alongside low doses and plasma levels in some PWEs. Conversely, there were examples of PWEs who did not respond or who reported no tolerability issues at high doses or plasma levels. **Conclusions:** To evaluate the individual therapeutic range and to better understand the influence of other drugs in cases where concomitant medications are used, the therapeutic drug monitoring of cenobamate may be useful. A general therapeutic range cannot be defined.

## 1. Introduction

Cenobamate (YKP 3089) (C_10_H_10_CIN_5_O_2_) ((IR)-1-(2-Chlorophenyl)-2-(2*H*-tetrazol-2-yl) ethyl carbamate) is a new antiseizure medication (ASM) that was synthesized, developed, and investigated in preclinical and clinical trials by SK Life Science Inc. It is a tetrazole alkyl carbamate derivative with one chiral center. Cenobamate is structurally related to, but different from, carisbamate, which was previously investigated as a potential ASM, but not pursued because one pivotal randomized controlled trial failed to show statistically significant superiority of efficacy over placebo [1,2,3,4].

In 2021, cenobamate was approved by the European Medicines Agency (EMA) for the adjunct treatment of adult patients with epilepsy (PWEs) with focal-onset seizures who did not respond in a satisfying way after an appropriate treatment with at least two established ASMs [5]. The approval was based on one proof-of-concept study [6], two randomized placebo-controlled trials [7,8], and one open-label safety study [9].

The pharmacological profile of cenobamate has been extensively investigated [5,10,11,12,13,14,15,16,17]. In brief, the key points are as follows.

The oral CNB absorption rate is 88% or higher and almost independent of food intake. The time to the maximum plasma concentration after oral intake (T_max_) varies between 0.8 and 4 h. The plasma protein binding rate is 60% and the distribution volume accounts for approximately 40–50 L. After single and multiple applications, the maximum plasma concentration C_max_ increases in a dose-proportional manner. Although the area under the curve (AUC) increases after single doses in a more-than-linear way, an almost dose-proportional increment was observed in the clinically relevant daily dose range between 50 mg and 500 mg. The oral clearance is low; it decreases from approximately 1.4 L/h to 0.50 L/h with increasing doses and ranges between 0.45 and 0.63 L/h at oral daily doses between 100 mg and 400 mg, which are recommended according to the summary of the product characteristics (SmPCs). The elimination half-life time T_1/2_ varies between 30 and 76 h with increasing doses. For daily doses between 100 mg and 400 mg, T_1/2_ is about 50–60 h. Considering this pharmacokinetic profile, a once-daily dosing in the evening is possible and, therefore, recommended. Under these conditions, plasma steady-state concentrations are reached after approximately 14 days.

Cenobamate is extensively metabolized in the liver. It undergoes glucuronidation via UGT2B7 and, to a lesser extent, via UGT2B4, and oxidation via CYP2E1, CYP2A6, CYP2B6, and, to a lesser extent, via CYP2C19 and CYP3A4/5.

Certain populations require special attention if the use of cenobamate is intended. The cenobamate plasma concentrations were shown to be increased in patients with a liver (plasma AUC = 2.1–2.3-fold higher) or renal (plasma AUC = 1.4–1.5-fold higher) impairment in pharmacokinetic studies [15,16]. The pharmacokinetics did not differ significantly based on gender or race, or among elderly (age > 65 years) compared with younger people (18–45 years) [16].

The precise mode of action has not yet been fully uncovered [4,5,11,15,16]. Two mechanisms have been described so far. One involves cenobamate reducing repetitive neuronal discharges through the inhibition of voltage-dependent sodium channels [5,7,10,16,18]. In particular, cenobamate enhances the rapid and complete inactivation of sodium channels and inhibits the non-activating component of the sodium current (I_NaP_) in hippocampal rat neurons in a concentration-dependent manner [3,5,6,10,11,16,18].

A second mechanism of action is related to γ-amino butyric acid (GABA) [5,8,12,16,18]. Cenobamate potentiates GABA-induced currents in acutely isolated CA3 pyramidal cells in a concentration-dependent manner. It enhances tonic GABA_A_ currents and shows a positive allosteric modulation of GABA-induced currents mediated by GABA_A_ receptors. This effect was similar for all of the tested GABA_A_ receptors containing six different alpha subunits (α_1_β_2_γ_2_ or α_2-6_β_3_γ_2_). The results showed that CNB acted as a positive allosteric modulator of high-affinity GABA_A_ receptors, activated by GABA at a site independent of the benzodiazepine binding site, and efficiently enhanced tonic inhibition in hippocampal neurons [10,18]. There is additional evidence that cenobamate interacts with both synaptic and extra-synaptic GABA_A_ receptors by exerting effects on both phasic (I_phasic)_ and tonic (I_tonic_) GABA_A_ currents [18].

Despite the limitations of fixed-dose placebo-controlled trials, such as relatively short maintenance periods, the phase-II trials already suggested that cenobamate might be a particularly effective ASM, even when compared to the results of similar successful studies with other recently investigated and approved ASMs [2,3,19].

The open-label extension data and some real-world studies support this view [20,21,22,23,24,25].

The Kork Epilepsy Center is one of the leading tertiary-referral epilepsy centers in Germany. We had the opportunity to join two of the pivotal trials with cenobamate [8,9] so that we were familiar with the practical handling of cenobamate and the perspective it offers. Since many of our patients suffer from long-lasting and difficult-to-treat epilepsies, we usually collect a high number of PWEs who are urgently waiting for a new treatment option. In addition, we run a therapeutic drug monitoring (TDM) laboratory that offers plasma-level measurements of almost all of the ASMs currently available. To define the clinical importance of the plasma levels of recently introduced ASMs, we regularly perform appropriate studies, most recently concerning perampanel [26,27]. With cenobamate, we were able to establish our own TDM essay in 2021 prior to the introduction of cenobamate on the market, the details of which are described in the methods section.

Here, we present our data on adult patients with focal-onset seizures who were treated with add-on cenobamate, and in whom we were able to assess cenobamate doses and the resulting plasma levels and to correlate them with efficacy and tolerability under plasma steady-state conditions.

## 2. Methods

This study is part of the ongoing CENKORK study, which is a prospective, single-center, open-label, non-interventional study in adult PWEs who were initiated with adjunct cenobamate after the introduction of cenobamate to the German market in June of 2021 and during the following year.

We measured the plasma levels of the participants whenever possible to determine the clinical usefulness of the TDM of cenobamate.

The following inclusion criteria were applied:

1. Initiation of add-on cenobamate between June 2021 and June 2022;
2. Stable antiseizure medication during the three months prior to the initiation of cenobamate;
3. Once-daily cenobamate dosing in the evening;
4. Venous blood collection in the morning;
5. Complete documentation of seizures and tolerability during the three months prior to the initiation of cenobamate;
6. Seizure frequency of at least one per month and no seizure-free intervals of at least four weeks during the preceding three months according to their self-reports and seizure calendars;
7. Recruitment for the CENKORK observational trial;
8. Adult age;
9. Informed consent;
10. TDM at the laboratory of the Kork Epilepsy Center;
11. Out-patient appointment and examination on the same day of the TDM by the first author (BJS).

The exclusion criteria were as follows:

1. Alteration in the daily dose of cenobamate within the preceding four weeks;
2. Alteration in antiseizure or other chronically applied co-medication within the preceding four weeks;
3. Incomplete documentation of seizure situation or tolerability;
4. History of non-epileptic seizures;
5. Status epilepticus during the preceding three months;
6. Renal or hepatic impairment, since the area under the curve may be increased in patients with a mild or moderate renal or hepatic impairment [13,15].

We assessed various demographic data, including age, gender, age at onset of epilepsy, seizure and epilepsy classification, etiology, previous ASMs and their number, the current ASM treatment, and the seizure frequency during the three months prior to the initiation of cenobamate.

The data were pseudonymized prior to the data analysis.

The PWEs were followed concerning the cenobamate titration and dosing, their seizure frequency and intensity, and adverse events (AEs). No fixed appointment schedules were used. The PWEs were followed on an individual basis. In every case, direct communication was possible so that dose adjustments could be performed.

Due to the unusually high efficacy of cenobamate according to the literature [2,5,7,8,9,15,16,21,22,23,24,25,27,28], we decided to analyze the data only in the PWEs who became completely seizure-free during the three-month period versus the non-responding PWEs, i.e., PWEs who experienced no effects on their seizure frequency or a seizure reduction of <50%. Thus, we excluded the so-called ≥50% responders to more accurately differentiate between unequivocal responders (seizure-free PWEs) and non-responding PWEs.

Although the long elimination half-life of cenobamate might allow plasma levels to be considered almost independently of the daytime and mode of intake [13,17], the plasma levels were only accepted when the blood samples were obtained in the morning, independently of food intake, which has no relevant impact on the absorption [5,15,16].

Cenobamate was titrated according to the schedule used in the phase-III trial by Sperling et al. (2020) [9], which is also recommended in the SmPC for the European Union. Thus, once-daily dosing was intended with 12.5 mg of cenobamate during titration weeks one and two, followed by dose increases to 25 mg during weeks three and four, 50 mg during weeks five and six, and 100 mg thereafter. At this timepoint, we regularly contacted the participants to communicate the subsequent strategy according to the efficacy and tolerability at week seven. In every case, we tried to dose as efficiently as possible on an individual basis, with 400 mg as the maximum dose according to the results of the pivotal trial by Krauss et al. (2020) [8] and the European SmPC.

The cenobamate levels were measured in our own certified TDM laboratory by means of liquid chromatography–mass spectrometry (LCMS).

A quantitative analysis of cenobamate (C_10_H_10_ClN_5_O_2_ → 267.67 g/mol) was performed using a mass spectrometric method. The ammonium adduct [M + NH_4_]^+^ was analyzed as an intense target ion. An isotope-labeled internal standard [^13^C_6_] cenobamate was used.

Sample preparation:

An amount of 100 µL of serum with 100 µL of an internal standard solution and 500 µL of methanol were mixed (for 10 s) and cooled (for 15 min). After centrifugation (10,000 rpm for 7 min), the supernatant was diluted with water (1:1).

The chromatographic conditions were as follows:
HPLC1260 Infinity II by Agilent Technologies (Waldbronn, Germany)ColumnPoroshell 120 EC-C 18 3.0 × 150 mm 2.7-Micron by Agilent TechnologiesColumn temp40 °CMobile phaseA: 5 mM Ammonium acetate (+0.2% formic acid) B: AcetonitrileIsocraticA/B → 55/45 (*v*/*v*)Flow rate0.4 mL/minInjection volume2 µL

The conditions of the mass spectrometer were as follows:
DetectorMSD G6125B by Agilent TechnologiesExtracted ionsCenobamate+[NH_4_]^+^: M = 285Internal standard [^13^C6] Cenobamate+[NH_4_]^+^: M = 291Fragmenter voltage80 VNebulizer pressure35 psigGas temperature200 °CDrying gas flow12 L/minVcap4000 V

The quantification of cenobamate in plasma allowed for the selective and sensitive determination of the compound. The linearity was investigated in a range from 0.1 µg/mL to 100 µg/mL. The measuring range was approved from 0.5 µg/mL to 50 µg/mL. The quantification limit was 0.5 µg/mL. The precision was <5%. The dilutions were unremarkable. Interference (by other ASMs) was not observed. Abnormalities in the stability of cenobamate in plasma (<5 days) were not detected. As an internal quality control, at least two controls are included in the approved measuring range for each measurement. These requirements are national guidelines (RILIBÄK) for every laboratory in Germany and therefore fulfill legal requirements. A calibration is carried out for each analysis batch. As no commercial control materials are available for new analysis methods, control material is prepared by weighing pure substance (from the company AlsaChim, Illkirch-Graffenstaden, France) and commercially available plasma (from the company Chromsystems, Gräfelfing, Germany). Until participation in a commercial interlaboratory comparison is possible, each parameter is compared externally with at least two samples twice a year with a laboratory based in Germany.

At the time of measuring the cenobamate plasma concentration, the additional medication, tolerability, and seizure situation were assessed. For the classification of efficacy, the preceding three months were compared with the three-month period prior to the initiation of cenobamate. An assessment of tolerability was performed on the day of the plasma level measurement by means of a direct interview and a clinical examination by one of the researchers (BJS).

The objectives of this investigation were as follows:

1. Is there a direct proportionality between the daily doses and the plasma levels as predicted by the literature, at least in lower to medium doses?
2. Are there differing levels of daily doses and plasma levels concerning efficacy?
3. Are there differing levels of daily doses and plasma levels concerning tolerability?
4. Is it possible from these real-world data to define the therapeutic range that is required to achieve an appropriate efficacy without a risk of AEs?

The statistical analyses performed were mainly descriptive in nature. Continuous endpoints were reported using means ± SD, and categorical data were reported using frequencies and percentages. The baseline characteristics and post-baseline measures are reported from the analyses, including the cenobamate dose and the plasma levels, separated by the status of the response (non-response vs. seizure freedom). Similarly, the status of AEs (present vs. absent) is reported. A linear regression and Pearson’s correlation analysis were also performed between the cenobamate daily dose and the plasma levels. The frequency and percentage of subjects with no response or seizure freedom are reported using the plasma concentration. An analysis of covariance (ANCOVA) was conducted to evaluate whether the serum concentration levels were different between seizure-free versus non-responding PWEs. Separately, a similar analysis was conducted to compare concentration levels in PWEs who experienced AEs versus those who did not. The ANCOVA models were adjusted for baseline seizure frequency as the covariate. A nominal two-sided alpha of 0.05 was used to identify any statistical differences.

This study was conducted according to the Declaration of Helsinki code of ethics. It was approved by the local ethical committee of the Medical Faculty at the University of Freiburg, Germany (No. 22-1139) and registered at the German registry of clinical studies, DRKS (DRKS-ID DRKS00030916). The participants gave their informed consent.

## 3. Results

The plasma levels were obtained from 101 PWEs; 36 of them were seizure-free and 65 were non-responding according to the definitions mentioned above. AEs were reported by 31 PWEs.

The demographic data are shown in Table 1.

The PWEs included in this study suffered from difficult-to-treat epilepsies with a mean duration above 20 years; a mean of around 10 previously used antiseizure medications, usually one or two concomitant ASMs; and a seizure frequency of more than 10 per month, despite the antiseizure baseline medication prior to cenobamate. No significant differences were apparent between the groups (see Table 1). Among the seizure-free PWEs, more subjects were male (57% male versus 43% female). The duration of epilepsy was slightly longer in non-responding patients and in PWEs who did not experience AEs. The seizure frequency prior to cenobamate was higher in PWEs who experienced AEs. However, these differences were well within the confidence intervals.

**Figure 1:**
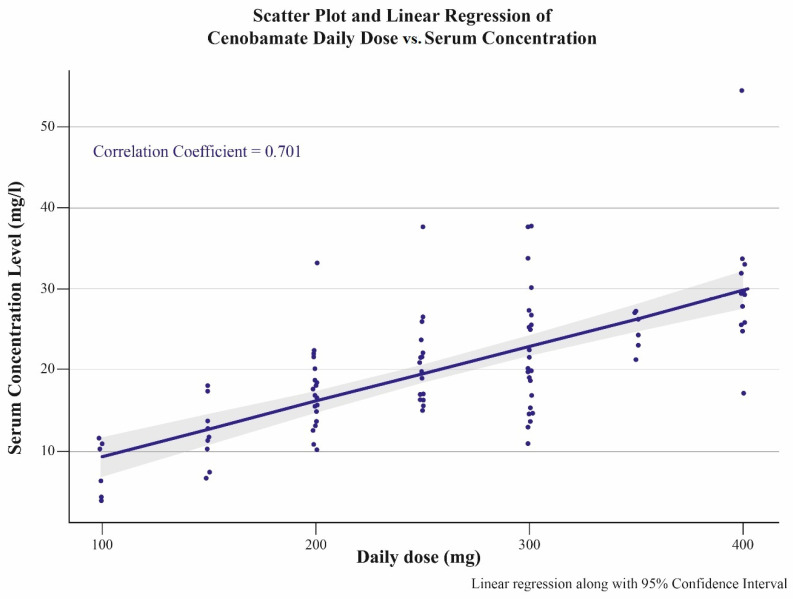
Correlation between daily doses of cenobamate (mg) and corresponding plasma levels (mg/L).

**Figure 2:**
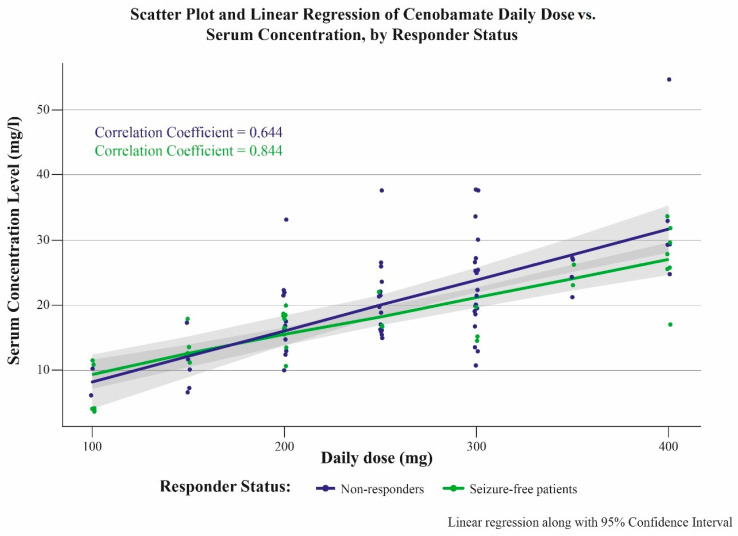
Correlation between daily doses of cenobamate (mg) and corresponding plasma levels (mg/L) in seizure-free and non-responding patients with epilepsy.

Independently of the efficacy and the tolerability, we found a strong and linear correlation between the daily cenobamate dose and the plasma levels (see Figure 1 and Figure 2). In the whole group, the correlation coefficient was 0.701; in seizure-free PWEs, it was 0.844, and in non-responders, it was 0.644.

## 4. Efficacy

The mean daily doses were 240.3 ± 102.0 mg and 260.8 ± 73.2 mg, respectively, with a dose range between 100 mg and 400 mg in both groups.

In seizure-free PWEs, the corresponding cenobamate plasma levels varied between 3.8 mg/L and 33.7 mg/L (mean of 17.8 ± 7.1 mg/L), compared to a range between 3.9 mg/L and 54.6 mg/L and a mean of 20.9 ± 8.8 mg/L in non-responders. The ANCOVA comparison of covariance did not show significant differences at the 0.05 level.

## 5. AEs

AEs were reported in 30.7% of PWEs (n = 31). They consisted of somnolence (n = 19, 61.3% of all PWEs who experienced AEs), followed by dizziness (n = 5, 16.1%) and ataxia (n = 3, 9.7%), along with single cases of a dazed state, diplopia, gynecomastia, increased liver enzymes, a depressive mood, a mood alteration, and tremor. The rate of AEs was 25% among seizure-free patients (n = 9) and 33.8% among the non-responding PWEs (n = 22). The daily cenobamate doses and plasma levels of PWEs who experienced AEs and those who did not experience them did not differ significantly. In PWEs who did not experience AEs, the mean daily dose was 257.1 ± 90.6 mg (100–400 mg); in PWEs who did experience AEs, it was 245.2 mg ± 70.0 mg (100 mg–400 mg). The corresponding mean cenobamate plasma levels were 19.5 ± 7.7 mg/L (3.8–37.8 mg/L and 20.4 ± 9.8 mg/L (3.9–54.6 mg/L), respectively. These results were not significantly different. When we addressed typical neurotoxic symptoms such as dizziness, ataxia, and blurred or double vision, the daily doses of cenobamate in the seven affected PWEs varied between 200 mg and 400 mg, with the plasma levels ranging between 13.6 and 54.6 mg/L. The concomitant medications were levetiracetam (n = 2), lamotrigine (n = 3), levetiracetam and oxcarbazepine (n = 1), and oxcarbazepine and phenobarbital (n = 1). The ANCOVA comparison of covariance did not reveal significant differences.

The complete results concerning the efficacy and tolerability are shown in Table 2.

## 6. Discussion

To the best of our knowledge, our study reports the first major data collection of cenobamate plasma levels in real life along with clinical correlations. In line with the literature [5,10,11,12,13,14,15,16,17], we found a linear correlation between the daily doses and the plasma levels, even at higher doses according to a dose-proportional increment. Thus, even under a varying concomitant ASM treatment, as in our study, the plasma levels corresponding with dose increments are sufficiently predictable.

TDM is an established tool to improve the quality of ASM therapy. Especially with first- and second-generation ASMs such phenobarbital, phenytoin, and carbamazepine, so-called therapeutic ranges have been defined and are still widely used [26]. The definition of a therapeutic or reference range is a drug-specific span between upper and lower limits that defines the range of doses resulting in probable antiseizure efficacy without dose-related AEs [29]. The concept of such a general definition has been questioned. Instead, the use of a more individualized therapeutic range has been proposed [30].

We established our LCMS assay early in 2021, around the time of the introduction of cenobamate to the market in Germany. Due to its long half-life, the once-daily dosing regimen in the evening, and a maximum time of four hours to achieve maximum plasma concentration (T_max_), relevant and interfering plasma level fluctuations after the blood sampling in the morning are very unlikely, similarly to the situation with perampanel [26]. At steady state, plasma level fluctuations are very limited [17]. Our experience supports this statement: we measured the daytime cenobamate levels under steady-state conditions in two of our in-patients at various timepoints (8 and 12 a.m., 4 p.m., and immediately prior to the intake of cenobamate in the evening). The fluctuations in the plasma levels were very slight and within a deviation of 10%. Therefore, we feel confident that our method was appropriate for addressing the study variables. Furthermore, we excluded any interfering modifications by concomitant medications or hepatic or renal impairments, and we used the cenobamate steady-state levels exclusively.

The definition of a so-called therapeutic range was not possible.

When comparing the data of seizure-free versus non-responding PWEs, no significant differences were found concerning either the doses or the plasma levels. The main reason for this is that the individual therapeutic ranges varied widely: seizure freedom as well as adverse effects were observed alongside low doses and plasma levels in some PWEs. Conversely, there were examples of PWEs who did not respond or reported no tolerability issues at high doses or plasma levels. The unusually high rate of seizure-free patients under cenobamate, which was already confirmed by many other reports [2,5,7,8,9,15,16,21,22,23,24,25,27,28], allowed us to adopt a rather radical approach that is unique in this study: To compare the results from seizure-free and non-responding patients and avoid the gray zone of so-called responders.

Our findings emphasize the recently proposed concept of an individualized therapeutic range [30] in favor of a general one: in seizure-free PWEs, it might be helpful to document not only the dose, but also the corresponding plasma level. The assessment of dramatic responders and non-responders did not provide any effect-specific general recommendations. Likewise, AEs and a good tolerability did not correlate with the plasma levels in general. Considering typical neurotoxic AEs such as dizziness, blurred vision, and ataxia, we also found a wide range of doses and plasma levels: the daily doses varied between 200 mg and 400 mg, and the plasma levels varied between 13.6 mg/L and 54.6 mg/L. Similar findings were found for other recently introduced ASMs such as lacosamide, brivaracetam or perampanel [26,31]. This reflects the wide interindividual range concerning the efficacy and tolerability of ASMs as adjuncts in real life with confounding factors like interfering pharmacokinetic and pharmacodynamic interactions with baseline ASMs or the individual susceptibility of PWEs. It is tempting to speculate whether the complex metabolism of cenobamate [14] may contribute to its individual tolerability based on individual metabolism and interfering factors of concomitant medication. Further and more detailed studies will be necessary to address this open question.

From the pivotal trials and the published real-world evidence, it has been claimed that combinations of cenobamate with sodium-channel-blocking agents (for pharmacodynamic reasons) or with clobazam (for pharmacokinetic reasons) are associated with a higher risk of AEs [32]. We could not confirm this assumption from our data because these AEs were more common under combinations with levetiracetam and lamotrigine, although the plasma level of the latter usually drops under the influence of cenobamate [5,11,15,16]. However, these findings were observations in small subgroups, and certainly require support from larger studies and cohorts.

It is tempting to speculate that responders at low doses and plasma levels may have been sensitive to the unique GABAergic mechanism of cenobamate at a binding site independent of the benzodiazepine binding site [10,18], especially if these PWEs were previously treated with an ASM with an effect on sodium channels comparable to cenobamate. The unique GABAergic mechanism of action in line with the sodium-channel-blocking activity of cenobamate that parallels other ASMs is shown in Table 3.

If such a specific effect occurs, the correlation with the dose and plasma levels might be less important.

Data on the clinical experience with cenobamate plasma levels are scarce. The levels of ≥50% responders in two of the pivotal studies [7,8] recorded plasma levels between 5 and 35 µg/mL in more than 95% of patients [34]. This is almost identical to the levels we found in seizure-free PWEs. However, we compared these findings with those in non-responding PWEs and did not find a clinically meaningful inter-group difference.

In clinical practice, it might be more important to collect and understand the plasma levels of concomitant ASMs that are influenced by cenobamate. This will be the objective of another study. The methodology will be more challenging because plausible conclusions will only be possible if PWEs are included with a stable concomitant medication over a longer period of time so that true cenobamate-induced alterations can be found and judged appropriately. This will require following the courses of individual PWEs over months, which was not performed in the study reported here.

## 7. Strengths and Limitations

Our study has the strength that the data were collected under identical circumstances. Data heterogeneity was reduced by the exclusive use of personal patients according to a monocenter design, and the plasma levels were only obtained at our center and investigated in our TDM laboratory.

The limitations result from the relatively low amount of data. However, the results were consistent, so we doubt that more data would have changed the key messages of our investigation.

## 8. Conclusions

Despite the close and linear correlation between the daily doses and plasma levels of cenobamate, a global therapeutic range could not be defined due to the highly individual reactions concerning the efficacy and tolerability at widely differing doses and levels. Nevertheless, TDM might be useful in well-defined clinical situations, such as investigations into the adherence or impact of potentially interacting concomitant medications or factors such as hepatic or renal impairment. The routine TDM of cenobamate is not recommended.

## Figures and Tables

**Table 1:** Demographic data.

	Seizure-Free Patients	Non-Responding Patients	No Adverse Events	Adverse Events
n	36	65	70	31
Age (years) (mean)	37.5 ± 15.9	37.9 ± 12.5	38.0 ± 14.0	37.3 ± 13.4
Age (years) (median)	32.5	35	33.5	39
Age (years) range	19–76	21–72	19–76	20–72
Female (n, %)	15 (43)	36 (55)	35 (50)	16 (52)
Male (n, %)	21 (57)	29 (45)	35 (50)	15 (48)
Duration of epilepsy (years) (mean)	22.5 ± 11.9	26.7 ± 14.5	26.7 ± 13.7	21.6 ± 13.3
Duration of epilepsy (years) (median)	20	25	23	15
Duration of epilepsy (years) (range)	7–55	1–56	6–56	1–48
Seizure frequency per month prior to cenobamate (in case of daily seizures limited to 30) (mean)	13.8 ± 12.8	13 ± 11.3	12.4 ± 11.5	15.2 ± 12.3
Seizure frequency per month prior to cenobamate (in case of daily seizures limited to 30) (median)	8	10	8	10
Seizure frequency per month prior to cenobamate (in case of daily seizures limited to 30) (range)	1–30	1–30	1–30	1–30
Number of previous antiseizure medications (mean)	9.6 ± 2.7	10.5 ± 4.5	10.0 ± 4.0	10.7 ± 3.8
Number of previous antiseizure medications (median)	10	10	10	10
Number of previous antiseizure medications (range)	4–15	4–27	4–27	5–19
Number of comcomitant antiseizure medications (mean)	1.5 ± 0.6	1.6 ± 0.7	1.6 ± 0.7	1.5 ± 0.6
Number of concomitant antiseizure medications (median)	1	2	2	1
Number of concomitant antiseizure medications (range)	1–3	0–3	0–3	1–3

**Table 2:** Results. Daily maintenance doses and plasma levels in seizure-free versus non-responding and in patients without and with clinical adverse events *.

	Seizure-Free Patients	Non-Responding Patients	No Adverse Events	Adverse Events
n	36	65	70	31
Seizure-free n (%)	36 (100)	0 (0)	27 (39)	9 (29)
Adverse events n (%)	9 (25)	22 (34)	0 (0)	31 (100)
Daily cenobamate dose (mean)	240.3 ± 102.0 mg	260.8 ± 73.2 mg	257.1 ± 90.6 mg	245.2 ± 70.0 mg
Daily cenobamate dose (median)	200 mg	250 mg	250 mg	250 mg
Daily cenobamate dose (range)	100–400 mg	100 mg–400 mg	100–400 mg	100–400 mg
Cenobamate plasma level (mean)	17.8 ± 7.1 mg/L	20.9 ± 8.8 mg/L	19.5 ± 7.7 mg/L	20.4 ± 9.8 mg/L
Cenobamate plasma level (median)	17.6 mg/L	19.9 mg/L	18.6 mg/L	18.4 mg/L
Cenobamate plasma level (range)	3.8–33.7 mg/L	3.9–54.6 mg/L	3.8–37.8 mg/L	3.9–54.6 mg/L

* The statistical comparison of the plasma levels between seizure-free PWEs and non-responders as well as between PWEs with and without clinical adverse events did not show significant differences at a 0.05 level.

**Table 3:** Mode of action of antiseizure medications (modified after [33]).

Mode of Action	Antiseizure Medication (Selection)
**Modulators of voltage-gated sodium channels**	
Increase in fast inactivation (transient sodium current: (I_NaT_)	Phenytoin, carbamazepine, oxcarbazepine, eslicarbazepine acetate, lamotrigine, possibly topiramate, zonisamide, rufinamide, brivaracetam
Increase in slow inactivation	Lacosamide
Block of persistent sodium currents (I_NaP_)	Cenobamate, lacosamide, carbamazepine, oxcarbazepine, eslicarbazepine acetate, lamotrigine, phenytoin, topiramate, valproate, gabapentin, cannabidiol
**Blockers of voltage-gated calcium channels (T-type)**	
High-voltage-activated	Phenobarbital; phenytoin, levetiracetam
Low-voltage-activated T-type (Ca_V_3)	Ethosuximide, methsuximide, eslicarbazepine acetate, possibly valproate
**Activators of voltage-gated potassium channels (K_V_7)**	Retigabine (ezogabine)
**Modulators of GABA-mediated inhibition**	
Allosteric modulators of GABA_A_ receptors	Phenobarbital, primidone, stiripentol, benzodiazepines, topiramate, felbamate, retigabine (ezogabine), cenobamate
Inhibitors of GAT1 GABA transporter	Tiagabine
Inhibitors of GABA transmaninase	Vigabatrin
Activators of glutamic acid decarboxylase	Possibly valproate, gabapentin, pregabalin
**Inhibitors of ionotropic glutamate receptors**	
Antagonists of NMDA receptors	Felbamate, topiramate, possibly valproate
Antagonists of AMPA receptors	Perampanel, phenobarbital, levetiracetam
**Modulators of the presynaptic release machinery**	
SV2A	Levetiracetam, brivaracetam
α2δ subunit of calcium channels	Gabapentin, pregabalin
Inhibitors of carbonic anhydrase	Acetazolamide, sulthiame, topiramate, zonisamide, possibly lacosamide
Serotonin-releasing agents	Fenfluramine
**Disease-specific modulators**	
Inhibitors of mTORC1 signaling	Everolimus

AMPA: α-amino-3-hydroxy-5-methyl-4-isoxazolepropionic acid, GABA: γ-aminobutyric acid, GAT: GABA transporter, mTORC1: mechanistic target of rapamycin complex 1, NMDA: N-methyl-D-aspartate, SV2A: synaptic vesicle protein 2A.

## Data Availability

Data were pseudonymized for the statistical analysis. Raw data are stored at the archive of the Kork Epilepsy Center.
